# Leveraging behavioral economics and implementation science to engage patients at risk for suicide in mental health treatment: a pilot study protocol

**DOI:** 10.1186/s40814-022-01131-y

**Published:** 2022-08-13

**Authors:** Gabriela Kattan Khazanov, Shari Jager-Hyman, Joseph Harrison, Molly Candon, Alison Buttenheim, Matteo F. Pieri, David W. Oslin, Courtney Benjamin Wolk

**Affiliations:** 1 Mental Illness Research, Education, and Clinical Center of the Veterans Integrated Service Network 4, Crescenz Veterans Affairs Medical Center, Philadelphia, PA USA; 2 Leonard Davis Institute of Health Economics, University of Pennsylvania, Philadelphia, PA USA; 3 Department of Psychiatry, University of Pennsylvania School of Medicine, Philadelphia, PA USA; 4 Center for Health Incentives and Behavioral Economics, University of Pennsylvania School of Medicine, Philadelphia, PA USA; 5 Philadelphia College of Osteopathic Medicine, School of Professional and Applied Psychology, Philadelphia, PA USA; 6 Department of Family and Community Health, University of Pennsylvania School of Nursing, Philadelphia, PA USA

**Keywords:** Suicide, Implementation, Treatment engagement, Treatment initiation, Behavioral economics, Science of behavior change, Mechanisms

## Abstract

**Background:**

Primary care is an ideal setting to connect individuals at risk for suicide to follow-up care; however, only half of the patients referred from the primary care attend an initial mental health visit. We aim to develop acceptable, feasible, low-cost, and effective new strategies to increase treatment initiation among at-risk individuals identified in primary care.

**Methods:**

We will conduct a multi-phase, mixed-methods study. First, we will conduct a chart review study by using administrative data, including medical records, to identify characteristics of primary care patients at risk for suicide who do or do not attend an initial mental health visit following a referral. Second, we will conduct a mixed methods study by using direct observations and qualitative interviews with key stakeholders (*N* = 65) to understand barriers and facilitators to mental health service initiation among at-risk individuals. Stakeholders will include patients with suicidal ideation referred from primary care who do and do not attend a first mental health visit, primary care and behavioral health providers, and individuals involved in the referral process. We also will collect preliminary self-report and behavioral data regarding potential mechanisms of behavior change (i.e., self-regulation and social support) from patients. Third, we will leverage these findings, relevant frameworks, and the extant literature to conduct a multi-arm, non-randomized feasibility trial. During this trial, we will rapidly prototype and test strategies to support attendance at initial mental health visits. Strategies will be developed with subject matter experts (*N* = 10) and iteratively pilot tested (~5 patients per strategy) and refined. Research will be completed in the Penn Integrated Care Program (PIC), which includes fourteen primary care clinics in Philadelphia that provide infrastructure for electronic referrals, patient communication, and data access.

**Discussion:**

We will leverage frameworks and methods from behavioral economics and implementation science to develop strategies to increase mental health treatment initiation among individuals at risk for suicide identified in primary care. This project will lead to an evaluation of these strategies in a fully powered randomized trial and contribute to improvements in access to and engagement in mental health services for individuals at risk for suicide.

**Trial registration:**

ClinicalTrials.gov Identifier: NCT05021224

## Background

Suicide is a major global public health problem. In 2018, there were approximately 800,000 suicide deaths worldwide [1] and over one million people attempt suicide each year [2]. The effects of suicide are far reaching, affecting family members, friends, and communities, and costing the USA more than $90 billion annually [3]. Despite increased attention to suicide as a significant public health concern, current suicide rates are approximately 30% higher than at the turn of the millennium [4]. Although evidence-based practices (EBPs) for suicide prevention exist and individuals are less likely to die by suicide if they engage in services [5–7], individuals at risk for suicide often have difficulty engaging in mental health treatment. To date, most research in this area has examined mental health treatment initiation following acute services for patients at risk for suicide [8, 9]. Studies have not yet developed or tested strategies to increase mental health treatment initiation among individuals at risk for suicide identified in primary care, even though a large proportion of these individuals do not go on to initiate mental health treatment following referral [10–12].

Primary care is a logical place to identify and connect individuals at risk for suicide to follow-up care because most adults visit primary care annually [13]. Approximately two thirds of people who die by suicide interact with a primary care clinician in the year prior to death [14]. While mental health screening and referral are common in primary care, even when patients are referred to a mental health provider within the same practice only half attend an initial visit [15, 16]. Strategies such as reminder calls and texts, motivational and informational interventions, and case management have only demonstrated small to moderate effects [17]. Even when these interventions are implemented, about 40% of patients do not initiate treatment [17], indicating the need for more effective methods for increasing treatment initiation.

**Fig. 1:**
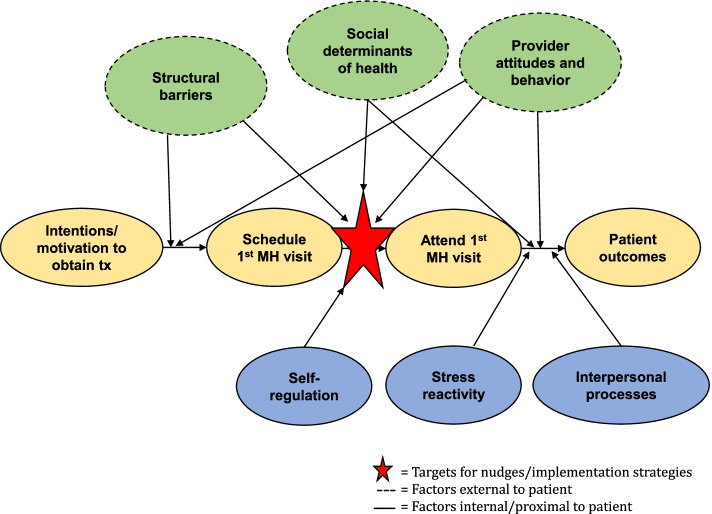
Conceptual model

Behavioral science theories and methods hold promise for improving patient engagement [18]. Behavioral economics, which combines classic economic theory with insights from psychology to understand common, predictable factors that influence decision making [19], can be leveraged to improve treatment engagement [20]. Applying behavioral insights effectively requires two steps. First, it is essential to understand the target problem, context, and behavioral and structural barriers and facilitators. Principles from implementation science provide guidance for achieving this understanding and can enhance knowledge of the ways in which organizational factors and stakeholder perspectives influence innovation adoption [21, 22]. Second, it is important to understand key mechanisms that underlie changes in behavior. The Science of Behavior Change (SOBC) provides an experimental therapeutic framework that guides the identification of mechanisms of change to better understand *how* and *why* certain strategies work [23]. Factors related to self-regulation (e.g., temporal discounting) and interpersonal and social processes (e.g., emotional social support) may impact the decision to initiate treatment. Thus, it is important to target putative mechanisms, such as temporal discounting and social support, when designing strategies and to attend to contextual factors that impact implementation success (see Fig. 1).

### Relevant frameworks

#### The EAST framework

As noted above, this study draws on principles from behavioral economics, implementation science, and experimental therapeutics. Behavioral economists have identified a wide range of heuristics in human decision making (i.e., cognitive biases), many of which contribute to poor health outcomes. The EAST Framework asserts that, to encourage behavior change and reduce people’s reliance on cognitive biases, the desired behavior should be Easy, Attractive, Social, and Timely [24]. The first of the EAST principles, make it **E**asy to change behavior, includes strategies such as utilizing default options (e.g., auto enrollment in 401k), reducing the effort required to perform an activity, and simplifying messaging. The second principle is to make the desired behavior **A**ttractive (e.g., use of attention-grabbing messaging and incentives). The third principle, make it **S**ocial, includes demonstrating that most people engage in the desired behavior, harnessing the power of networks, and encouraging people to express commitment to others. The final principle, make it **T**imely, focuses on careful consideration of when to implement strategies, such as providing prompts when people are most likely to be receptive or engaging in collaborative planning for action.

Four steps are recommended to apply these insights [24]: (1) define the outcome (i.e., increase attendance at first mental health visit), (2) understand the context (i.e., what stops people from attending), (3) build the intervention (i.e., develop implementation strategies), and (4) test, learn, and adapt (e.g., evaluate implementation strategies and engage in rapid prototyping). Principles from both experimental therapeutics and implementation science can be leveraged to execute these steps with maximum effectiveness and inform our conceptual model (Fig. 1). For example, in order to understand the context (step 2), it is essential to examine *why* people have difficulty engaging in a desired behavior (i.e., the context) and *why* or *how* an intervention works to change behavior (i.e., mechanisms). Principles and methods from implementation science, such as conducting contextual inquiries to identify barriers and facilitators to evidence-based practice implementation, can be applied to better understand the context and determinants affecting treatment engagement.

#### The science of behavior change (SOBC) framework

The SOBC Common Fund Program, which applies an experimental therapeutics approach to behavior change, has identified key domains of mechanisms relevant to multiple health behaviors and clinical outcomes that informed our conceptual model and measurement strategy [23]. Mechanisms, critical to advancing the science of implementation, refer to “processes that are responsible for change” or the specific way by which implementation strategies affect implementation success and patient outcomes. Identification of mechanisms can lead to more efficient, effective, and tailored strategies based on the practice of interest and the implementation context [25].

Self-regulation and interpersonal and social processes, two SOBC domains, may mediate the relationship between engagement strategies and treatment initiation. Within the domain of self-regulation, temporal discounting—the cognitive process of discounting rewards that are not immediate—may be a relevant mechanism (e.g., choosing to turn off the alarm clock to gain the immediate reward of sleeping versus attending an initial therapy appointment, which promises the future reward of symptom reduction). A potential strategy to target this mechanism might be a financial incentive to attend treatment sessions [26]. A second putative SOBC mechanism is social-emotional processes (e.g., emotional social support), one type of interpersonal, or social process. Caring Contacts, for example, are brief, non-demanding letters or texts expressing care or concern that have been associated with decreases in suicidal ideation and deaths and may target this mechanism [27, 28].

## The promise of rapid prototyping

To address this public health crisis, we must expedite the rate at which innovations to engage individuals at risk for suicide in treatment enter routine practice [29, 30]. Traditionally, intervention effectiveness is tested by expensive, lengthy trials, followed by dissemination and implementation activities. Rapid prototyping involves a series of brief, rigorous tests to optimize operations in early-study stages. Using rapid prototyping increases the likelihood that interventions tested in trials will be acceptable, feasible, and effective, thereby shortening the process of developing and implementing interventions. Industries outside of health care commonly use this approach to learn quickly and “de-risk” decision-making prior to a large rollout [31, 32].

Leveraging the work of the Penn ALACRITY (Advanced Laboratories for Accelerating the Reach and Impact of Treatments for Youth and Adults with Mental Illness) Center for transforming mental health delivery through behavioral economics and implementation science [33], we will develop, pilot, and optimize strategies to increase treatment initiation for persons at risk for suicide and accelerate the pace at which promising strategies enter routine care. We will first develop strategies in collaboration with a team of experts in suicide, implementation science, and behavioral economics. We will then iteratively pilot test and refine these preliminary strategies using rapid prototyping. Through this process, we will uncover additional barriers and facilitators to implementing strategies that will allow us to further refine and optimize them prior to testing them in a future randomized trial.

### Aims

**Fig. 2:**
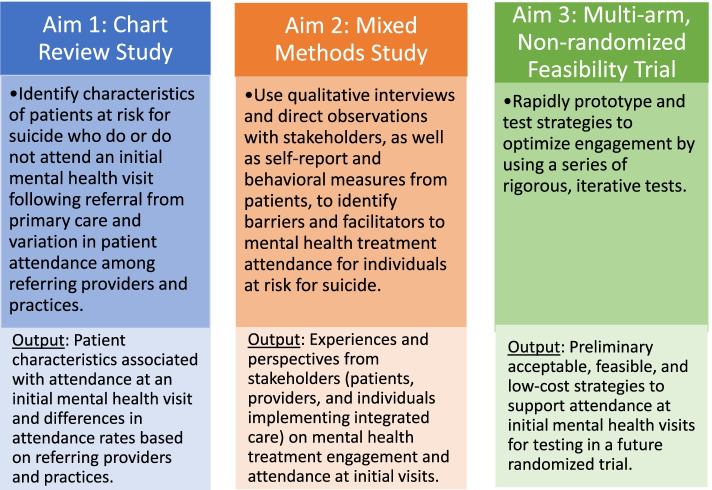
Study aims and design for multiphase, mixed-methods study

The main objective of this study is to develop acceptable, feasible, low-cost, and effective strategies that increase patients’ treatment initiation (i.e., attendance at a first mental health visit) following identification of suicide risk in primary care. To complete this objective, we will partner with a large and diverse health system to conduct a multiphase, mixed-methods study (see Fig. 2). We will first conduct a chart review study to identify characteristics of patients at risk for suicide who do or do not attend an initial mental health visit following a referral from primary care and variation in patient attendance among referring providers and practices (Aim 1). We will then conduct a mixed methods study by using qualitative interviews and direct observations with stakeholders to identify barriers and facilitators to mental health treatment attendance for individuals at risk for suicide (Aim 2). In accordance with an experimental therapeutics approach to behavior change, we will also collect preliminary data on mechanisms of action that may impede treatment attendance (i.e., self-regulation and social support) from patients using self-report and behavioral measures [34–37]. Finally, using procedures from implementation science [38] and behavioral economics [39], we will leverage the information gleaned from Aims 1 and 2, relevant theories and frameworks (e.g., EAST [24];), and the extant literature to develop preliminary strategies to support attendance at first mental health visits. We will then conduct a multi-arm, non-randomized feasibility trial to rapidly prototype and test strategies to optimize engagement by using a series of rigorous, iterative tests (Aim 3). To maximize generalizability, we will develop strategies for increasing attendance in both collaborative care and outpatient specialty mental health.

## Method

### Setting

In 2018, a collaborative care program was developed through a partnership between Penn’s Department of Psychiatry and Primary Care Service Line and is referred to as the Penn Integrated Care (PIC) program (a detailed description is provided by Wolk and colleagues [40];). Fourteen primary care clinics in Philadelphia and the surrounding counties that range in size and include family medicine and internal medicine practices have PIC services embedded in their clinics. These practices provide infrastructure for electronic referrals, patient communication, and data access. All practices use a sophisticated electronic health record (EHR) system and patient registries that enables the capture of episodes of care and billing for services using Collaborative Care billing codes [41]. A master’s level mental health clinician is embedded within each practice and delivers traditional Collaborative Care services in collaboration with a consulting psychiatrist [42], as well as brief evidence-based interventions, including behavioral activation, motivational interviewing, and problem-solving therapy, for those with mild to moderate mental illness [40]. The PIC model also includes a centralized “Resource Center” staffed by bachelors-level mental health intake coordinators who are supervised by licensed clinicians. This Resource Center assesses, triages, and refers primary care patients in need of mental health services to the health system and community providers.

#### Aim 1 (chart review study)

Aim 2 is to identify the characteristics of patients at risk for suicide who do or do not attend an initial mental health visit following referral from primary care and variation in patient attendance among referring providers and practices.

#### Sample

The sample will consist of adults referred for mental health services from primary care in PIC practices, including all Medicare, Medicaid, and commercial insurance enrollees. Based on preliminary data, we anticipate that our study sample will include 916 adults with a score of ≥1 on the PHQ-9 suicide item who were referred to PIC between 2018 and mid-2021. By comparison, there were 2470 adults with a score of ≥1 on the PHQ-9 suicide item who were referred to specialty care and/or did not have insurance coverage for PIC.

#### Procedure

We will use administrative data, including medical records and insurance claims data, to identify which patient characteristics (e.g., diagnoses, demographics) are associated with attendance at mental health visits and examine variations in patient attendance among referring providers and practices. Our primary data source will be medical records; when available, we will also use insurance claims data. These analyses are exploratory.

#### Measures

We will rely primarily on medical records to examine treatment engagement among patients with suicidal ideation or behaviors. Treatment engagement will be measured by attendance at a mental health visit following a referral. For a subset of patients, we will review insurance claims data to examine whether they utilized specialty care outside of Penn Medicine. The patients selected will depend on access to insurance claims and approval to analyze these claims.

#### Medical records

Medical records will be de-identified and sent securely to our study team by program leadership and the Data Analytics Center at Penn Medicine. They will include patient demographic and clinical information, including age, gender, race/ethnicity, insurance type, any utilization of care at Penn Medicine (e.g., specialty care, hospitalizations), prescriptions, patient acuity (on a 1–4 scales), and behavioral health screenings (e.g., PHQ-9 for depression, GAD-7 for anxiety [43, 44];).

#### Insurance claims

Insurance claims will provide similar demographic and utilization data as medical records but will additionally include any care utilization occurring outside of Penn Medicine. We anticipate that insurance claims will be accessible for Medicaid enrollees from Philadelphia and/or enrollees of one major commercial insurer.

#### Analyses

We will compare characteristics across (1) patients who screen positive for suicidal ideation (have a score of ≥1 on item 9 of the PHQ-9, which is administered at least annually to all Penn patients), and attend the first mental health visit following referral [43], to (2) patients who screen positive for suicidal ideation and do not attend the first mental health visit following referral. We will detect differences in group means using *t* tests, then use logistic regressions to identify characteristics that are associated with engagement.

Our equation of interest is **A**i = α + **X**iβ + εI, where **A** is a binary variable that indicates whether patient *i* attended their initial follow-up appointment after the suicide screening. The vector **X** includes covariates, including age, gender, and race/ethnicity. The unit of analysis is the patient level. We will cluster standard errors at the clinic level and assess model appropriateness and fit using the relevant diagnostics.

We will also compare the two groups’ service use by examining outpatient, inpatient, and pharmacological treatment in the year prior to screening. For patients with accessible insurance claims, we will measure service utilization after the PHQ-9 screen to track mental health treatment engagement outside of Penn Medicine [43]. This will serve as a validity check of medical records to measure engagement because the claims will capture relevant primary or specialty care accessed outside of Penn Medicine.

#### Aim 2 (mixed methods study)

Aim 2 is to use qualitative interviews and direct observations with stakeholders, as well as self-report and behavioral measures from patients, to identify barriers and facilitators to mental health treatment attendance for individuals at risk of suicide.

#### Sample

Following the initial inspection of the EHR data in Aim 1, we will identify patients referred from Penn primary care to mental health services following a positive screen for suicidal ideation on the PHQ-9 who did or did not attend their scheduled mental health visit over the past 3 months [43]. All patients must (1) be 18 years and older, able to communicate in English, and able and willing to provide informed consent and (2) present with elevated suicidal ideation per item 9 of the PHQ-9 (item score ≥ 1) completed during a primary care visit. Exclusion criteria include (1) an imminent risk of suicide (i.e., plan with the method and intent to act) and (2) currently experiencing a psychotic episode requiring emergency services and/or precluding abilities to provide informed consent.

Additionally, we will interview primary care and behavioral health providers, as well as individuals involved in the implementation of integrated care at Penn. We will include Penn primary care providers who have referred one or more patients with a positive screen for suicidal ideation for mental health services over the past 3 months. Behavioral health providers will include those practicing at Penn and in community clinics to which Penn patients are routinely referred. Once we identify patients and providers, we will randomly select individuals to invite to participate in qualitative interviews. Individuals involved in the implementation of integrated care at Penn include leaders in Primary Care and Psychiatry, supervisors of behavioral health providers, and mental health intake coordinators. All providers, leaders, and intake coordinators must (1) be 18 years and older, able to communicate in English, and able and willing to provide informed consent and (2) be employed in an appropriate primary care or behavioral health role.

We anticipate conducting a total of 65 interviews including with patients who do (*n* = 12) and do not (*n* = 12) attend a first mental health visit, primary care providers (*n* = 12), behavioral health providers (*n* = 12), intake coordinators (*n* = 5), and leaders (*n* = 12). While we anticipate conducting 65 interviews, we will continue until saturation is achieved.

#### Procedure

Guided by EAST, we will use observation and semi-structured qualitative interviews with key informants to understand stakeholder experiences and perspectives around mental health treatment engagement. Interviews will include questions about barriers and facilitators of treatment initiation and attendance. For patient stakeholders, we also will assess self-regulation and social support with self-report and behavioral measures. Participants will be compensated $30 an hour for participating. Finally, we will observe intake coordinators in the Resource Center as they assess and schedule patients with mental health providers. Although we are underpowered to detect significant effects, we hypothesize that lower self-regulation and lower social support will predict patient non-attendance at initial mental health visits.

### Measures (Table 1)

#### Self-regulation

We will measure self-regulation, specifically temporal discounting, using two SOBC-recognized measures: (1) The Kirby Delay-Discounting Task (KDDT) [34] and (2) The Consideration of Future Consequences (CFC) Scale [36].

**Table 1:** Alignment of measures with conceptual model

Construct(s)	Measure/data source
Self-regulation	Kirby Delated Discounting Task [34]; Consideration of Future Consequences Scale [36]
Interpersonal processes (social support)	The Multidimensional Scale of Perceived Social Support [37]
Structural barriers: provider attitudes	Qualitative interviews: providers, intake coordinators, leaders
Structural barriers: perceptions of provider attitudes	Qualitative interviews with patients
Provider behavior	Observations
Social determinants of health: attendance at first mental health visit	Data from EHR

The KDDT is a behavioral task that assesses the tendency to devalue rewards as they become more temporally remote (known as *delay-discounting*) that has demonstrated reliability and validity [34, 35, 45]. The KDDT has 27 items that provide two choices for each question: an immediate reward or a larger but delayed reward (e.g., $19 today or $25 in 53 days). Acceptable test-retest reliability has been established at 5 weeks (*r* = .77) and 1 year (*r* = .71; 37).

The CFC scale is a 12-item self-report measure of consideration of future consequences or forward-thinking behavior [36]. Each CFC item (e.g., “Often I engage in a particular behavior in order to achieve outcomes that may not result for many years”) is rated on a 1-5 Likert-type scale ranging from “extremely uncharacteristic” to “extremely characteristic.” The total score ranges from 12 to 60, with higher scores indicating greater consideration of future consequences. The CFC has demonstrated strong internal consistency (Cronbach’s alpha .80–.86) and acceptable test-retest reliability (*r* = .72) [36].

#### Interpersonal processes (emotional social support)

We will measure perceived emotional social support using the 12-item Multidimensional Scale of Perceived Social Support (MSPSS) [37]. The MSPSS, a self-report measure recognized by the Research Domain Criteria Project (RDoC), is rated on a 1–7 Likert-type scale ranging from “Very Strongly Disagree” to “Very Strongly Agree” in response to each statement (e.g., “I get the emotional help and support I need from my family”). The total score ranges from 12 to 84, with higher scores indicating greater perceived social support. Internal consistency (Cronbach’s alpha = .88) and test-retest reliability (*r* = .85) are well established [37]. Divergent validity of the MSPSS has also been established; the measure has been found to be inversely correlated with measures of depression and anxiety [46].

#### Structural barriers and provider attitudes (qualitative interviews)

Individual, semi-structured interviews will be conducted to understand cognitive biases and contextual factors that may impact treatment engagement broadly and attendance at initial mental health visits in particular. We developed interview guides for each stakeholder group to ensure uniform inclusion and sequencing of topics and to allow for valid comparison across interviews. The first section asks about general views related to mental health services, including perceptions of (1) what constitutes mental health care, (2) why mental health care is or is not important, (3) barriers and facilitators to attending mental health visits, (4) use of and experience with mental health services, and (5) engagement strategies used or recommended (for providers, intake coordinators, leaders).

The second section asks about relevant cognitive biases, including (1) what is appealing and satisfactory about mental health care, (2) what is unappealing and unsatisfactory about mental health care, and (3) perceived expectations of others related to mental health care. Finally, patients will be asked to recount the screening, referral, and appointment process and, if they attended a mental health visit, steps implemented or supports utilized to attend. For patients who did not attend, we will similarly ask them to recount any steps they attempted to take and barriers they encountered.

#### Provider behavior (observations)

Study staff will spend one full week observing intake coordinators in the centralized mental health assessment, triage, and referral management center (i.e., PIC Resource Center). Observations will include detailed field notes of processes and procedures to understand leverage points for increasing engagement.

### Analyses

#### Qualitative analysis

We will upload interviews and field notes into NVivo for data management and analysis. Analysis will be guided by an integrated approach that includes identification of a priori attributes of interest (i.e., EAST principles) and modified grounded theory, which provides a rigorous, systematic approach to identifying emergent codes and themes [47]. This approach uses an inductive process of iterative coding to identify recurrent themes, categories, and relationships. After initial exploration of data, a comprehensive coding scheme will be developed and applied to all data. The product will be a fine-grained descriptive analysis of the challenges to engagement in mental health services, including issues related to attendance at first visit following referral. Two members of the research team will separately double code a sample of transcripts to assess reliability of the coding scheme. Disagreements will be resolved through team discussion.

#### Quantitative analysis

To explore potential mechanisms, we will use descriptive statistics to examine the distribution of self-report (CFC and MSPSS) and behavioral task (KDDT) scores. We will use one-way ANOVAs to examine the relationships between attendance and scores on the CFC, MSPSS, and KDDT. These analyses are exploratory to inform future trials; the study will not be powered to detect effects.

#### Mixed methods analysis

We will integrate quantitative data from Aim 1 and interviews using the following taxonomy: the structure is Quan—QUAL, the function is to expand upon the quantitative findings to understand the *process* of engagement as experienced by stakeholders, and the process is connecting [47]. To integrate the quantitative and qualitative methods, we will follow the NIH guidelines for best practices [48]. We will use the quantitative data to identify patterns in the qualitative data. We will enter quantitative findings into NVivo as attributes of each participant (i.e., did/did not attend; referral to PIC or community; KDDT, CFC, and MSPSS scores). These attributes will be used to categorize and compare important themes among subgroups.

#### Aim 3 (multi-aim, non-randomized feasibility trial)

Aim 3 is rapidly prototype and test strategies to optimize engagement by using a series of rigorous, iterative tests.

In Aim 3, we will develop preliminary strategies to support attendance at initial mental health visits and iteratively test and refine these strategies through rapid prototyping. Secondarily, we will explore the role of two potential mechanisms, self-regulation and social support, in the use of engagement strategies and attendance at initial mental health visits.

#### Sample

Each strategy will be tested with approximately 5 patients. All patients must be (1) 18 years and older and able to communicate in English and (2) present with elevated suicidal ideation on item 9 of the PHQ-9 (item score ≥1) completed during a primary care visit. In addition, patients must not (1) currently be experiencing a psychotic episode requiring emergency services and/or precluding their ability to provide informed consent, (2) have a documented diagnosis of dementia in the past 2 years, (3) have primary care provider notes demonstrating that participation is not indicated, and (4) have already received a study engagement strategy following a different primary care visit. We will leverage the infrastructure of the Resource Center to recruit from the pool of patients who were referred for a mental health intake following a positive screen for depression or suicide risk in primary care based on the EHR. This provides a mechanism for recruiting patients while also ensuring that there is appropriate clinical screening and expertise to triage individuals in need of acute services. We anticipate that 25 patients will complete the temporal discounting and social support behavioral task and self-report measures, though additional patients may receive the strategies. Attendance data will be available from any individual who receives a strategy as part of routine care quality assurance.

#### Procedure

A team of experts in implementation science (*n* = 2), behavioral economics (*n* = 2), suicide (*n*= 2), and primary care and behavioral health (*n* = 4, total *N* = 10) will convene a half-day retreat to guide Aim 3 activities and develop strategies for rapid prototyping. This team will review key barriers and facilitators to attendance at initial mental health visits identified in Aims 1 and 2 and then generate strategies for facilitating treatment initiation that address barriers and leverage facilitators. The EAST framework will guide the selection of strategies [24]. We will also favor simple, established strategies [49] with freely available resources (e.g., Caring Contacts templates from Now Matters Now) to promote scalability [50], but will also consider other potential implementation strategies as indicated [31]. We will explore both low- and high-tech (e.g., EHR integration) strategies to maximize relevance across contexts. Based on the literature, previous work, and our conceptual model, we hypothesize that strategies that counteract delayed discounting tendencies (e.g., incentives) and foster emotional social support (e.g., Caring Contacts) will be needed [27].

We will then use rapid prototyping to test and refine the strategies identified by the team of experts [31], a process that has been applied successfully at Penn over the past 6 years [51–54]. The goal of rapid prototyping is to determine the best strategies for broader implementation in a subsequent trial. Prototype strategy designs will be validated through rapid-cycle tests in this real-world context and in a manner that is quick and inexpensive (see Table 2 for examples). This will allow us to fail fast and learn quickly. As we deploy strategies and learn from our experiences, we will adapt and iterate to refine promising strategies.

**Table 2:** Implementation strategy crosswalk

Examples of barriers	EAST constructs [24]	Potential strategies	Mechanisms
Effort required to attend treatment outweighs motivation	Make it attractive (e.g., attention grabbing messaging, incentives)	Financial incentives [55]	Counters delayed discounting (i.e., self-regulation)
Hopelessness, social disconnection	Make it social	Caring contacts [27, 28]	Enhances perceived social support (i.e., interpersonal processes)
Forgetting; rescheduling an appointment that no longer works can be challenging to navigate	Make it easy to change behavior and timely (e.g., plan for action)	Test interactive texting with study staff vs. automated texting to confirm/change appointment if needed [56]	Reduces structural barriers

Strategies will be rolled out in the PIC Resource Center over 6 months. During this period, strategies will be implemented by an embedded mental health intake coordinator as part of routine care. Consent to receive strategies is, therefore, not required. However, because participants will complete measures assessing acceptability and feasibility of these strategies, as well as temporal discounting and social support, we will employ an IRB-approved modified consent procedure prior to implementing strategies that includes a waiver of *written* consent. The modified consent procedure will consist of (1) a brief description of the study purpose, procedures, and potential risks; (2) notification that the strategy is part of a research project for which participation is voluntary; and (3) instructions on declining further participation. This modified approach will allow us to circumvent potential confounds associated with limiting a trial examining engagement to those who provide active, written informed consent to receive engagement strategies. By waiving the requirement of traditional, written informed consent, we will minimize the likelihood of a biased sample and maximize the validity and generalizability of our results [57, 58]. We anticipate that each strategy will be tested with ≥5 patients, but this is flexible; additional tests will be implemented as needed.

#### Measures

After testing each strategy, we will quantitatively assess patients’ perceptions of its feasibility, acceptability, and appropriateness using three brief, validated questionnaires: Acceptability of Intervention Measure (AIM), Intervention Appropriateness Measure (IAM), and Feasibility of Intervention Measure (FIM), that were developed to better evaluate and monitor implementation outcomes [59]. Each questionnaire consists of four items (total of 12 items) rated on a 1–5 Likert-type scales ranging from “Completely disagree” to “Completely Agree.” The total score ranges from 4 to 20 for each measure, with higher scores indicating greater acceptability, appropriateness, or feasibility, respectively. Reliability was demonstrated with Cronbach’s alphas of .85 for acceptability, .91 for appropriateness, and .89 for feasibility, and test-retest reliability was demonstrated with correlations of .80 for acceptability, .73 for appropriateness, and .88 for feasibility.

Patients will then complete the temporal discounting and social support behavioral task and self-report measures (the KDDT, CFC, and MPSS) described in Aim 2.

#### Analyses

Descriptive statistics will be used to describe feasibility, acceptability, and appropriateness of strategies as assessed with the AIM, IAM, and FIM. We will also use logistic regressions to examine the impact of engagement strategies on attendance at initial mental health visits using attendance information derived from the administrative data (did/did not attend). These analyses will provide us with a preliminary estimate of the effectiveness of each engagement strategy. Finally, we will use descriptive analyses to examine scores on the temporal discounting and social support self-report and behavioral tasks, as well as relationships between scores, engagement strategies, and attendance at initial mental health visits. These analyses are exploratory to inform mechanisms to investigate in future trials.

### Power analyses

Consistent with recommendations for pilot studies, we have not conducted power analyses for Aims 1–3. We instead focus on exploring the data at this stage [60], as well as examining the feasibility and acceptability of strategies to support attendance at initial mental health visits. Analyses will be oriented towards identifying promising strategies and mechanisms to focus on in subsequent studies.

## Discussion

Primary care has been identified as a crucial setting for connecting individuals at risk for suicide to follow-up care, yet only half of patients referred to mental health treatment by primary care providers attend an initial visit [15, 16]. Surprisingly, no studies have yet developed or tested strategies to increase mental health treatment initiation for individuals at risk for suicide identified in primary care. This study aims to use frameworks and methods from behavioral economics, implementation science, and experimental therapeutics to develop acceptable, feasible, low-cost, and effective strategies to increase treatment initiation in this population. To do this, we will conduct a multiphase, mixed-methods study to identify characteristics of patients at risk for suicide who do and do not initiate mental health treatment, examine barriers and facilitators to treatment initiation among patients at risk for suicide, and rapidly prototype and test strategies to increase engagement.

This study has multiple strengths. First, we will partner with a large, diverse health system to identify barriers and facilitators to treatment initiation and develop and test potential solutions. Second, we will leverage rich administrative data to capture clinical characteristics and mental healthcare utilized both inside and outside the health system. This approach addresses key limitations of medical record use, which does not capture utilization outside the health system, and insurance claims, which lack verified diagnoses [61]. Third, unlike most studies of patient engagement, we will both quantitatively and qualitatively examine engagement strategies. Fourth, we will concurrently assess the perspectives of multiple stakeholder groups, including providers, patients, and health system leaders, which is a valuable approach that is rarely taken. Fifth, we will integrate methods and measures from behavioral economics, implementation science, and experimental therapeutics to develop and refine implementation strategies and elucidate generalizable mechanisms of engagement. Finally, we will rapidly prototype and test engagement strategies, which increases the potential for successful implementation and decreases the research-to-practice timeline.

Despite these strengths, the current study also has several limitations. In particular, the study will be conducted in a single, well-resourced health system with an existing integrated care program; the extent to which the results will generalize to other health systems is unknown. However, the demographic and economic diversity of the Penn Medicine patients may offset threats to external validity. In addition, given our focus on establishing feasibility and acceptability of implementation strategies, our sample size for testing strategies will be small. As such, statistical power will be limited, precluding our ability to draw firm conclusions about the strategies’ effectiveness. Furthermore, although we will examine associations between mechanisms of behavior change (i.e., self-regulation and social support) and treatment initiation among individuals at risk for suicide, our small sample size will preclude us from testing whether these mechanisms mediate the relationship between engagement strategies and treatment initiation. The current research, however, will be pivotal in paving the way for larger, confirmatory trials testing the effectiveness of these strategies and relevance of these mechanisms of behavior change. Finally, the current study is focused on treatment initiation rather than ongoing treatment engagement. Given that this is a formative trial, we chose to operationalize treatment engagement in this way to maximize parsimony, feasibility, and generalizability of our findings. It is possible, however, that effective strategies may differ for initial and ongoing treatment engagement. Examining strategies to improve ongoing treatment engagement is beyond the scope of the current trial, but we aim to broaden our operationalization of engagement in a subsequent larger, adequately powered trial.

In sum, this study will produce novel information about factors that enable and prevent primary care patients at risk for suicide from following up on mental health treatment. It will also produce a menu of strategies likely to increase treatment initiation among these individuals, along with potential mechanisms that may account for changes in treatment initiation. We anticipate that this project will lead to future studies testing the strategies developed to increase treatment initiation in a randomized, multi-site implementation trial. Our ultimate goal is to improve access to and engagement in mental health services for individuals at risk for suicide. This goal has the potential to reduce mortality among the majority of individuals with suicidal ideation who regularly attend primary care but not specialty mental health care.

## Data Availability

NA.

## Abbreviations

AIM: Acceptability of Intervention Measure
ALACRITY: Advanced Laboratories for Accelerating the Reach and Impact of Treatments for Youth and Adults with Mental Illness
ANOVA: Analysis of variance
CFC: Consideration of Future Consequences scale
EAST: Easy, Attractive, Social, and Timely
EBPs: Evidence-based practices
EHR: Electronic health records
FIM: Feasibility of Intervention Measure
GAD-7: General Anxiety Disorder-7
IAM: Intervention Appropriateness Measure
KDDT: Kirby Delay-Discounting Task
MSPSS: Multidimensional Scale of Perceived Social Support
NIH: National Institutes of Health
PHQ-9: Patient Health Questionnaire-9
PIC: Penn Integrate Care Program
RDoC: Research Domain Criteria Project
SOBC: Science of Behavior Change
